# Complete Genome of *Bacillus megaterium* Podophage Page

**DOI:** 10.1128/genomeA.00332-14

**Published:** 2014-04-17

**Authors:** Mariana S. Lopez, Mary K. Hodde, Karthik R. Chamakura, Gabriel F. Kuty Everett

**Affiliations:** Center for Phage Technology, Texas A&M University, College Station, Texas, USA

## Abstract

*Bacillus megaterium* is a Gram-positive, spore-forming saprophytic inhabitant of diverse environments. It is a reservoir for industrial chemical production and is emerging as a model organism for studying sporulation and protein localization. Here, we introduce the complete genome of Page, a novel podophage infecting *B. megaterium*.

## GENOME ANNOUNCEMENT

*Bacillus megaterium* is widespread in the environment and has many industrial uses (1). Because of its utility, the use of *B. megaterium* as a model organism for protein production has increased. Due to their tractability, plasticity, and rapid replication rates, bacteriophages present one of the most efficient opportunities for studying microbes. Here, we present the complete genome of the novel *B. megaterium* phage Page.

Bacteriophage Page was isolated from a soil sample collected in College Station, TX. Phage DNA was sequenced using 454 pyrosequencing at the Emory Georgia Research Alliance (GRA) Genome Center (Emory University, GA). Trimmed FLX Titanium reads were assembled to a single contig at 326.8-fold coverage using the Newbler Assembler version 2.5.3 (454 Life Sciences) at the default settings. The contig was confirmed to be complete by PCR. Genes were predicted using GeneMarkS (2) and corrected using software tools available on the Center for Phage Technology (CPT) Portal (https://cpt.tamu.edu/cpt-software/portal/). Transmission electron microscopy was performed at the Microscopy and Imaging Center at Texas A&M University.

Page is a podophage with a 39,874-bp circularly permuted genome containing 50 coding sequences. It has a G+C content of 40.7% and a coding density of 96%. Twenty-four coding sequences were annotated based on InterProScan results and BLASTp searches (3, 4). Ten genes were cross-referenced to existing hypothetical genes, and the remaining 16 were classified as hypothetical novel genes. Page has a broad host range and infects *B. megaterium* Km Sp^-^, DSM1804, DSM321, QM B1551, PV361, and WSH-002 strains.

Page contains genes encoding proteins related to a variety of functions (DNA replication, biosynthesis, packaging, transcriptional regulation, morphogenesis, and lysis). The genes for DNA replication proteins include those encoding a DnaB-DnaD replication protein, a DnaA replication initiator/ATPase protein, and several double-stranded and single-stranded DNA-binding proteins. Page also encodes an HNH homing endonuclease. A dUTPase that functions to maintain low dUTP levels in the cell was found (5). The head-to-tail joining protein, TerS, and TerL were identified. The TerL of Page has homology with TerLs of phages with *pac*-type head-full packaging. Page also encodes a putative transcriptional regulator protein and an RNA polymerase sigma factor.

Several tail protein genes were identified (encoding tailspike, tail fiber, and a lytic tail protein). The tailspike protein contains a pectin lyase domain hypothesized to be involved in biofilm degradation (6). The tail fiber gene was identified by its carbohydrate-binding H-type lectin domain (7). The lytic tail protein is predicted to have a transglycosylase and peptidase domain. An interesting find was a gene encoding an FtsK/SpoIIIE protein. These proteins act as ATP-dependent DNA translocases in Gram-negative and Gram-positive bacteria, respectively (8). How they are involved in the phage life cycle has yet to be determined.

### Nucleotide sequence accession number.

The genome sequence of phage Page was contributed as accession no. KF669655 to GenBank.
